# Behavioral Dynamics of Zebrafish Under Hydrodynamic Stimuli Induced by Magnetic Microactuator in Microfluidics

**DOI:** 10.3390/bios16090480

**Published:** 2026-09-01

**Authors:** Dineshkumar Loganathan, Pu-Hsiang Wang, Chia-Yuan Chen

**Affiliations:** 1 Department of Mechanical Engineering, National Cheng Kung University, Tainan 701, Taiwan; dinemon27@gmail.com (D.L.);; 2 Department of Power Mechanical Engineering, National Tsing Hua University, Hsinchu 300, Taiwan

**Keywords:** magnetic microactuator, hydrodynamic stimuli, zebrafish, memory, microfluidics

## Abstract

Behavioral investigation in zebrafish is essential for understanding adaptive responses, where learning represents a key process influenced by external stimuli. The applied stimulus plays a critical role in shaping such responses, therefore making physiologically relevant stimulation strategies important. Hydrodynamic stimuli represent one such modality, providing a natural and non-invasive means of activating mechanosensory responses in aquatic organisms, thereby enabling behavioral manipulation in microfluidic environments. To address this, a microfluidic assay was developed to generate controlled hydrodynamic environments by employing multiple S-shaped magnetic microactuators (SMMAs). Further, motions of these SMMAs were independently controlled to produce spatiotemporally varying vortical flow fields, enabling flow-induced transportation of zebrafish larvae. Flow dynamics were characterized by employing micro-particle image velocimetry (µPIV). Compared to the control condition, transportation time under microactuator-assisted guidance was significantly reduced, with a maximum improvement of 94.3% observed for a representative target zone. Building on this validated transport capability, training-dependent behavioral adaptation was quantified using latency under repeated hydrodynamic-training, where a reduction of 82.7% was achieved. Post-training assessment further demonstrated short-term retention of the acquired behavioral response followed by progressive extinction. These findings demonstrate that the proposed paradigm serves as a foundational behavioral assay leveraging hydrodynamic cues for studying adaptive responses in microfluidics.

## 1. Introduction

Learning is one of the fundamental components of human cognitive behavior and plays an essential role in processes such as decision-making, behavioral adaptation, and information retention. To investigate the biological and neural mechanisms underlying these cognitive processes, laboratory model organisms have been widely employed [1], through which controlled experimental studies of learning and memory have been conducted. Particularly, in recent years, small aquatic model organisms such as zebrafish larvae have emerged as powerful systems for investigating neural mechanisms underlying cognitive behavior [2,3,4]. Zebrafish larvae exhibit well-characterized neural circuitry, genetic accessibility, and transparent bodies that enable direct observation of behavioral responses and neural activity. These features have allowed zebrafish to become a widely adopted vertebrate model for studying sensory processing, learning behavior, and memory formation under controlled laboratory conditions [5]. Traditionally, behavioral assays designed to evaluate learning behavior in zebrafish have relied on visual [6,7], chemical [8,9], acoustic [2,4,10], or electrical stimuli [11,12]. Though these approaches have provided valuable insights into cognitive responses, the stimuli employed in many experimental platforms often differ significantly from the natural environmental cues experienced by aquatic organisms. As a result, the behavioral responses observed under such artificial conditions may not fully represent the naturally occurring sensory interactions encountered by aquatic species. For instance, in several behavioral conditioning studies, electric shock stimuli were employed to induce aversive learning responses in zebrafish [13], through which avoidance behavior and memory retention were evaluated. However, such externally imposed stimuli do not naturally occur in aquatic environments, and therefore, the measured learning and memory responses may not fully reflect natural cognitive behavior. Thus, the identification and implementation of stimuli that more closely resemble natural environmental cues are considered important for enabling more physiologically relevant investigations of learning behavior in aquatic model organisms.

In natural aquatic environments, organisms frequently rely on hydrodynamic signals to sense surrounding conditions, detect obstacles, and interpret the presence or motion of other organisms [14,15]. Hydrodynamic stimuli generated by fluid motion constitute an important sensory modality for many aquatic species. It has been reported that such stimuli were detected through mechanosensory systems, such as the lateral line system in fish [16,17], which enables the perception of local flow disturbances and water vibrations. Consequently, hydrodynamic cues play a critical role in navigation, predator avoidance, schooling behavior, and environmental exploration. In previous behavioral and physiological studies, several experimental approaches were employed to generate hydrodynamic disturbances for investigating zebrafish responses. For instance, rapid water movements were generated by employing silica capillaries connected to computer-controlled piezoelectric actuators, through which localized lateral water displacements were produced to stimulate mechanosensory responses in zebrafish larvae. Similarly, controlled minute water motions (mWMs) were applied to examine hydrodynamic sensing and mechanotaxis behaviors [18]. In addition, vortex-induced flows generated by stir bars placed within experimental chambers were utilized to induce forced swimming and rheotaxis behavior, enabling the investigation of stress responses and cortisol dynamics [19]. Other studies employed swirling protocols or vortex mixing in confined tubes and containers to create strong mechanosensory water disturbances capable of triggering physiological responses [20]. Although these techniques have provided valuable insights into hydrodynamic sensing and its related behavioral responses in zebrafish larvae, many of these approaches rely on bulk flow generation or mechanical agitation that limits spatial programmability and localized stimulus control. Therefore, the development of dynamically controllable systems capable of generating localized hydrodynamic stimuli remains important for enabling more physiologically relevant investigations of learning behavior in aquatic model organisms.

Recent advances in microrobotics and microfluidic technologies have opened new opportunities for generating controlled hydrodynamic fields at small spatial scales. Magnetically actuated microdevices [21,22,23,24,25,26,27], in particular, have emerged as promising tools for creating localized flow disturbances within microfluidic environments [28,29,30]. These devices can be wirelessly actuated and precisely manipulated by employing external magnetic fields, enabling programmable motion without the need for physical connections or integrated power sources [31,32,33,34,35,36]. Specifically, various microrobot architectures have been developed for microfluidic flow manipulation, including helical microrobots inspired by bacterial flagella [37,38] and artificial swimmer-type microrobots [39,40] capable of translational and rotational locomotion. In several studies, helical swimmers were magnetically rotated to generate propulsion and surrounding flow fields [41], while swimmer-type microrobots were employed to induce fluid stretching and folding processes that enhance mixing and transport within microscale environments [42]. However, many of these approaches primarily rely on navigation-based locomotion of microrobots to generate fluid disturbances. For applications that require hydrodynamic stimulation of living organisms, direct contact between mobile microrobots and biological samples may introduce mechanical interference or potential damage. Therefore, alternative strategies that employ stationary or anchored microrobotic structures capable of inducing programmable and controllable fluid motions have attracted increasing attention. In such configurations, magnetic actuation can be employed to drive localized swinging motions, partial rotations, or full rotational actuation of microrobotic structures while maintaining a fixed spatial position, thereby generating well-defined vortical flow fields without requiring translational movement of the device. This stationary actuation strategy enables non-contact hydrodynamic interaction with nearby organisms while maintaining precise spatial confinement of the generated flow. In this context, magnetically responsive micro-architectures that remain spatially fixed while generating programmable hydrodynamic stimuli provide a promising platform for investigating controlled organism–flow interactions within microfluidic environments.

In this work, a behavioral microfluidic assay was developed by employing S-shaped magnetic microactuators (SMMAs) to generate spatiotemporally varying hydrodynamic stimuli for investigating training-dependent behavioral adaptation in zebrafish larvae. The microactuators were magnetically actuated to induce rotational motion under anchored conditions within the microfluidic channel, thereby producing localized vortical flow disturbances in the surrounding fluid. Through the coordinated positioning and independent actuation of multiple microactuators, spatially programmable flow fields were generated to enable flow-induced transportation of zebrafish larvae toward designated regions of the microchannel. The effectiveness of the proposed system was first evaluated by examining larval transport performance across multiple target zones, through which navigation pathways were assessed based on transport efficiency. In particular, the least preferred target zone was selected to minimize inherent navigation bias and to establish a stringent condition for subsequent behavioral evaluation. Under this condition, repeated hydrodynamic guidance was employed as a training mechanism, in which larvae were exposed to controlled flow cues that directed their movement toward the selected region. The resulting training-dependent behavioral response was quantified using latency as a measure of navigation efficiency. Following repeated training, retention of the acquired behavioral response was further evaluated through a post-training assessment to examine its short-term persistence and subsequent extinction. The presented framework, therefore, establishes a microrobotic platform through which controlled hydrodynamic interactions can be harnessed to systematically investigate training-dependent behavioral adaptation and short-term behavioral retention in aquatic organisms.

## 2. Experimental Sections

### 2.1. Materials and Fabrication of S-Shaped Magnetic Microactuators

The S-shaped magnetic microactuators (SMMAs) introduced in this study were fabricated by utilizing a composite material system designed to provide magnetic responsiveness and mechanical stability suitable for microfluidic environments. The actuator structure consisted of a polydimethylsiloxane (PDMS) (SYLGARD™ 184, Dow Silicones Corporation, Midland, MI, USA) matrix integrated with hard magnetic particles. Meanwhile, the base polymer was prepared by mixing PDMS and the curing agent at a 10:1 weight ratio [43,44]. It should be noted that the polymer PDMS was chosen for its biocompatibility, optical transparency, and flexibility [45,46,47,48]. To impart magnetic functionality, neodymium–iron–boron microparticles (MQP-15-7, Magnequench International, Singapore) with an average diameter of 5 µm were dispersed into the uncured polymer at a weight ratio of 4:1 [49]. The resulting mixture was thoroughly homogenized and subsequently introduced into a precision-fabricated mold defining the S-shaped actuator geometry prepared using a micro-milling process.

### 2.2. Electromagnetic Actuation System and Control

Magnetic actuation of the microactuators was achieved by employing a custom-built electromagnetic coil array positioned beneath the microfluidic device [50]. Each solenoidal coil was constructed by winding 30 turns of copper wire around a soft-iron core with a height of 14 mm and a diameter of 1 mm. The coil array was mounted at a vertical separation of 1 mm below the microchannel to ensure effective magnetic coupling. A representative coil supplied with an input current of 2 A generated a magnetic flux density of 4.0 mT at the coil surface. The actuation system was integrated with a data acquisition unit (NI cDAQ-9174, National Instruments Corporation, Austin, TX, USA) and a programmable DC power supply (GPR-3510HD, Good Will Instrument Co., Ltd., New Taipei City, Taiwan), through which the current input to individual coils was precisely regulated. A LabVIEW-based interface was employed to control coil activation sequences and timing, thereby enabling programmable generation of dynamic magnetic fields. Under this configuration, selected coils were continuously maintained in the energized state to anchor the microactuators, while surrounding coils were sequentially activated in a defined temporal order to induce controlled rotational motion. Through this stepwise modulation of the magnetic field, both clockwise and counterclockwise rotations were achieved, enabling independent and spatially selective actuation of microactuators for generating localized hydrodynamic flow fields.

### 2.3. Microfluidic Device and Imaging Setup

The microfluidic device employed in this study consisted of a structured channel network incorporating an initial loading zone and multiple target zones interconnected through predefined pathways, fabricated by employing CNC milling and micro-casting techniques compatible with PDMS-based microfluidics. The device was designed to enable controlled hydrodynamic transport and guidance of zebrafish larvae through spatially distributed microactuators. The experimental setup included the electromagnetic actuation system, a programmable control unit, and an optical imaging system for real-time observation. Both the motions of SMMA and larval behavior were recorded by employing a CCD camera (WAT-902H ULTIMATE, Watec Co., Ltd., Tsuruoka, Yamagata, Japan) equipped with a macro lens (AF Micro-NIKKOR 60 mm f/2.8D, Nikon Corporation, Tokyo, Japan), enabling high-resolution visualization of flow-induced larval responses within the microchannel.

### 2.4. Micro-Particle Image Velocimetry (µPIV) Analysis

The hydrodynamic flow fields generated by the rotational actuation of the microactuators, including velocity, shear rate, and vorticity, were quantified by employing µPIV. Fluorescent polystyrene tracer particles with a diameter of 8 µm (Microgenics, Inc., Fremont, CA, USA) were suspended in deionized water and introduced into the microchannel using a syringe-driven flow system [51]. Particle motion under actuation was recorded using a high-speed camera (NR4-S2, Integrated Design Tools, Inc., Pasadena, CA, USA) mounted on a fluorescence microscope (BX60, Olympus Corporation, Tokyo, Japan). During µPIV measurements, the SMMAs were rotationally actuated at 8 Hz for an acquisition duration of 5 s, corresponding to 40 complete rotational cycles. The recorded image sequences were processed using Dynamic Studio 2015 (Dantec Dynamics A/S, Skovlunde, Denmark), where velocity fields were computed using a multi-pass adaptive cross-correlation algorithm with interrogation window refinement from 32 × 32 pixels to 16 × 16 pixels with 50% overlap [52,53,54,55]. Outlier vectors were removed using RMS-based validation [56,57,58,59,60,61,62]. The velocity fields obtained throughout the 5 s acquisition period were ensemble-averaged over the repeated rotational cycles to obtain a representative velocity field of the hydrodynamic response generated by the continuously rotating SMMAs. From the obtained velocity components, the corresponding shear rate and vorticity fields were subsequently calculated based on their spatial gradients. The resulting shear rate and vorticity distributions were also ensemble-averaged over the acquired rotational cycles to obtain representative spatial distributions of these hydrodynamic parameters. Thus, the velocity, shear rate, and vorticity fields were collectively employed to characterize the magnitude, spatial variation, and rotational characteristics of the hydrodynamic environment generated through rotational SMMA actuation.

### 2.5. Zebrafish Larvae Handling

Wild-type AB strain zebrafish larvae (*Danio rerio*) of 5 d.p.f. were obtained from the zebrafish facility at National Cheng Kung University and maintained under controlled laboratory conditions (Experimental work employing zebrafish larvae was reviewed and approved by the Institutional Animal Care and Use Committee (IACUC) of National Cheng Kung University, Tainan, Taiwan (Approval ID: IACUC-114163)). The larvae were kept at 28 ± 1 °C in a temperature-controlled environment under a 14:10 h light–dark cycle with illumination near 500 lux [63,64,65]. The rearing medium was refreshed every 24 h, and water quality parameters were maintained within standard ranges, including a pH of 7.0–7.5 and conductivity of 500–550 μS/cm [66,67,68,69]. Further, the larvae were maintained in filtered system water with dissolved oxygen levels above 5 mg/L and essential physicochemical conditions. The larvae were fed paramecia twice daily prior to experiments. For hydrodynamic guidance and behavioral conditioning experiments, larvae at 6 d.p.f. were transferred into the initial zone of the microfluidic device. Further, the 6 d.p.f. zebrafish larvae employed in the experiments had an average body length of 3.62 ± 0.50 mm and a maximum body width of 0.57 ± 0.03 mm. The minimum width of the constricted region within the microfluidic channel was 1.80 mm, which was more than three times larger than the maximum body width of the larvae. Therefore, the larvae were able to pass through (swim) the constricted regions longitudinally without observable mechanical obstruction. All larvae employed in the experiments exhibited comparable body sizes together with normal morphology and swimming behavior, and no larvae were excluded based on body size. This handling protocol ensured consistent physiological conditions and reliable behavioral responses during experimentation.

### 2.6. Behavioral Training and Post-Training Retention Assessment

A total of eight zebrafish larvae were employed for the behavioral training experiments, and the same eight larvae were individually followed throughout the complete experimental sequence from the pre-training assessment through all 10 training cycles. Each larva underwent 10 consecutive training cycles under controlled SMMA actuation, and latency was employed as the quantitative parameter to evaluate the training-dependent behavioral response. The same larvae were followed across the pre-training, fifth-cycle, and 10th-cycle measurements to maintain consistency throughout the repeated training process. Following completion of the 10th training cycle, the same eight larvae were further employed for the post-training retention assessment. The SMMA was removed, and the retention of the trained behavioral response was evaluated at 3 min intervals over a total duration of 15 min, corresponding to measurements at 3, 6, 9, 12, and 15 min. The memory-extinction parameter, termed freezing, was employed to quantify the degree of retention of the trained behavioral response over the post-training period. Freezing was determined based on the latency measured at each post-training time point relative to the latency obtained at the end of the 10th training cycle and the corresponding pre-training latency.

### 2.7. Temperature Measurement During Electromagnetic Actuation

Possible local temperature variations associated with electromagnetic actuation were experimentally evaluated by monitoring the temperature at different locations within the microfluidic channel. The temperature was measured by employing an infrared thermometer with laser targeting equipment, TN-433L (Hila International, Taipei City, Taiwan), with a measurement range of −38 to 365 °C. Further, temperature measurements were performed from 0 to 120 s under the same electromagnetic actuation conditions employed in the larval guidance experiments. Measurements were obtained at multiple locations within the microfluidic channel to determine the spatial and temporal variation in temperature during the complete actuation period. The corresponding temperature profiles are presented in the Supplementary Information.

### 2.8. Paramecia Culture

Paramecia employed in this study were obtained from the institutional animal facility (the Institutional Animal Care and Use Committee (IACUC) of National Cheng Kung University, Tainan, Taiwan (Approval ID: IACUC-114163) and maintained under controlled laboratory culture conditions to ensure a stable and reproducible population. The organisms were cultured in 100 mL containers and supplied with nutritional sources consisting of oatmeal and yeast to support sustained growth. The feeding process was performed at intervals of 5–6 days to maintain water quality and organism density. During feeding, oatmeal was finely crushed and directly introduced into the culture medium, whereas yeast was first diluted in water and then added in small volumes of 0.1–0.2 mL to prevent aggregation and avoid rapid degradation of the medium. The culture environment was maintained at a temperature range of 20–22 °C with a pH between 6.5 and 7.5, and gentle aeration was provided to ensure sufficient oxygenation and to minimize sedimentation [70,71,72]. The condition of the culture was monitored visually, where a clear medium indicated depletion of nutrients and the need for replenishment, while a slightly turbid appearance indicated adequate Paramecia density. To preserve long-term culture viability, fresh medium obtained from the animal facility was supplemented at regular intervals of two weeks. In the present work, Paramecia were utilized as a live food source for zebrafish larvae.

### 2.9. Statistical Analysis

All statistical analyses were performed to evaluate differences in larval transport behavior and behavioral response metrics across the tested conditions. Owing to the continuous nature of the measurements and the absence of extreme outliers, the data were considered to follow a normal distribution, thereby enabling the use of parametric statistical tests. For comparisons between two independent groups, an independent-sample *t*-test was applied. This test was employed to compare the success rate of larvae reaching the target zones and their transportation time between the microactuator-assisted and control conditions.. For behavioral analysis, a one-way analysis of variance (one-way ANOVA) was applied to evaluate differences across multiple time intervals. Specifically, one-way ANOVA was employed to analyze the latency values quantified at target zone T5 under pre-training and at different stages during the training process. All quantitative results were expressed as mean ± standard deviation, and statistical significance was denoted using standard notation, where * represents *p* < 0.05.

## 3. Results and Discussion

### 3.1. Design and Fabrication of the SMMA-Enabled Microfluidic Platform

**Figure 1:**
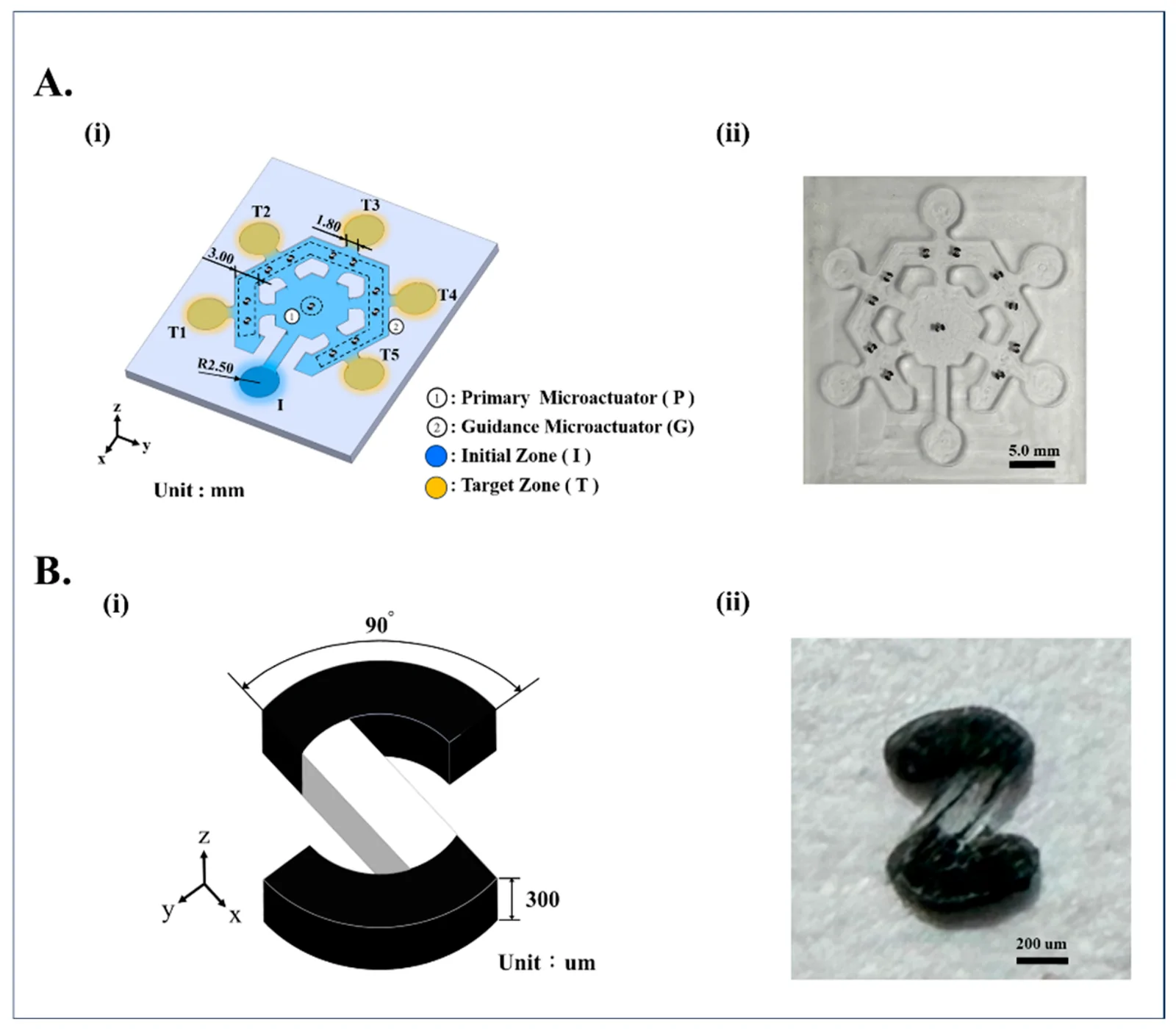
Design and realization of the SMMA-enabled microfluidic platform. (**A**) The employed microfluidic platform. (**i**) Schematic illustration showing the spatial arrangement of the initial zone (I), five target zones (T1–T5), primary microactuator (P), and guidance microactuators (G). (**ii**) Optical image of the fabricated microfluidic device. (**B**) S-shaped magnetic microactuator (SMMA). (**i**) Three-dimensional schematic illustrating the geometric design and dimensions of the SMMA. (**ii**) A representative optical image of the fabricated SMMA.

A microrobotic platform was developed to generate programmable hydrodynamic stimuli for investigating the behavioral responses of zebrafish larvae. As illustrated in Figure 1(Ai), the platform consisted of a microfluidic chamber integrated with multiple S-shaped magnetic microactuators (SMMAs), an initial zone (I), and five spatially distributed target zones (T1–T5). A primary microactuator (P) was positioned near the initial zone, whereas multiple guidance microactuators (G) were distributed along the transportation pathways. Although the SMMAs shared an identical geometric structure and actuation principle, their functional roles were defined according to their spatial locations. The primary microactuator was employed to generate a localized hydrodynamic disturbance for initiating larval displacement, whereas the surrounding guidance microactuators were selectively actuated to regulate the subsequent direction of larval movement toward the designated target zones. Meanwhile, the five target zones were distributed around the microfluidic chamber to enable multidirectional transportation of freely swimming zebrafish larvae. Since the initial orientation and swimming direction of the larvae within the microfluidic environment could vary, the transportation capability was required to remain effective irrespective of the direction of the designated target relative to the larval position. In accordance with this, T1–T5 were spatially distributed around the chamber to span the complete range of transportation directions. The selection of these zones was therefore primarily determined by the microfluidic geometry and the requirement to demonstrate multidirectional larval transportation rather than by a predetermined behavioral preference for a particular target zone. Furthermore, the spatial configuration enabled different pairs of guidance SMMAs to be selectively actuated to generate localized hydrodynamic fields for directing the larva toward the corresponding target location. A representative optical image of the fabricated microfluidic platform is presented in Figure 1(Aii). The design and geometry of the SMMA are presented in Figure 1(Bi). The magnetic microactuator was designed with an S-shaped curved architecture to generate vortical flow structures under rotational magnetic actuation. The curved configuration enabled effective interaction between the SMMA and the surrounding fluid while the actuator remained spatially confined within the microfluidic environment. A representative optical image of the fabricated SMMA is shown in Figure 1(Bii), demonstrating the realization of the designed S-shaped structure. Thus, the designed geometry was successfully realized as an individual magnetic microactuator for subsequent integration and actuation within the microfluidic platform.

**Figure 2:**
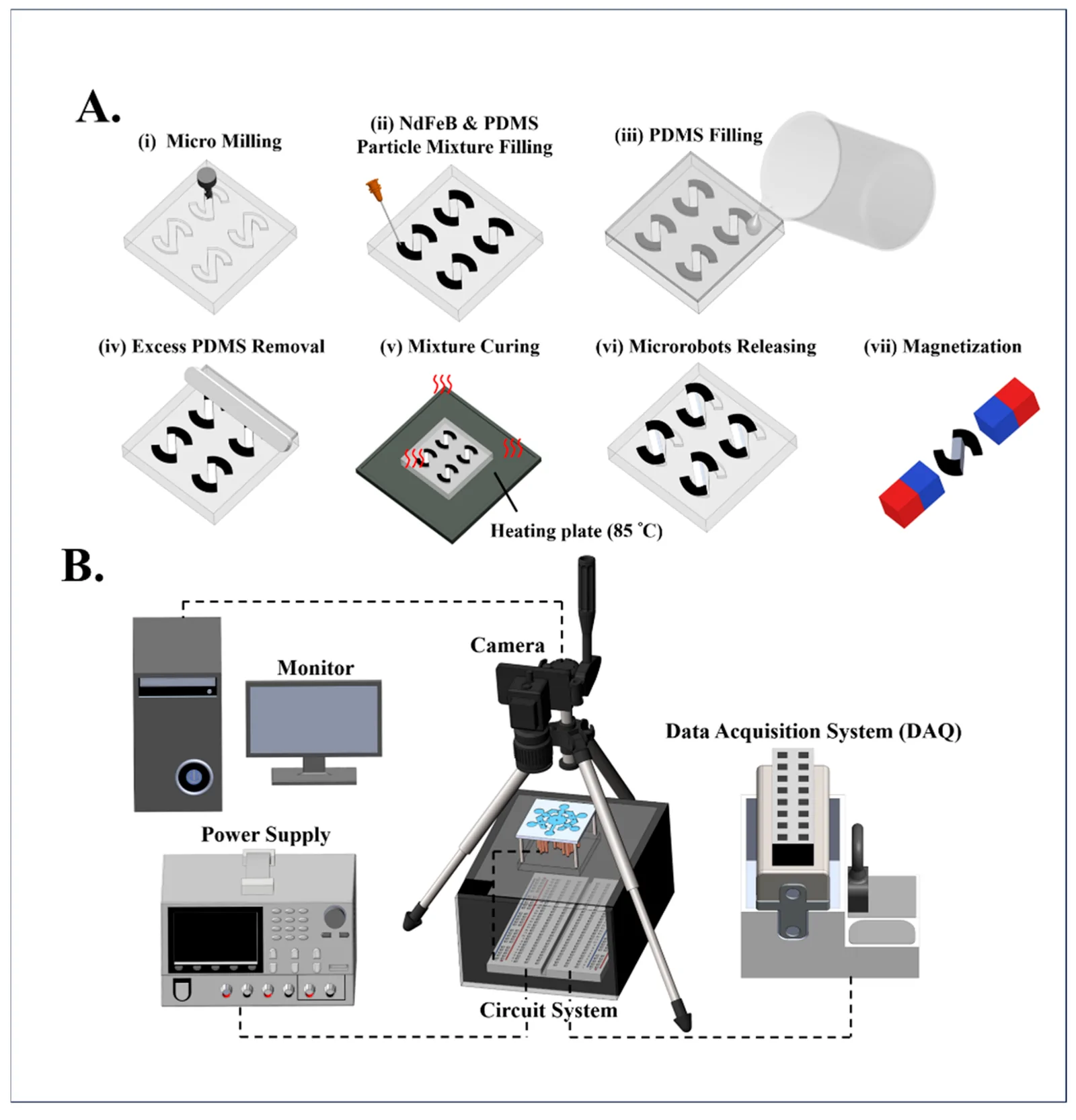
Fabrication process and experimental setup of the SMMA-enabled microfluidic platform. (**A**) Stepwise fabrication procedure of the SMMA showing (**i**) preparation of the S-shaped mold cavities using precision micro-milling, (**ii**) filling of the mold cavities with the NdFeB magnetic particle and PDMS composite mixture, (**iii**) PDMS filling, (**iv**) removal of excess PDMS, (**v**) thermal curing at 85 °C, (**vi**) release of the fabricated SMMAs from the mold, and (**vii**) magnetization of the SMMAs. (**B**) Experimental setup employed for electromagnetic actuation and real-time observation of the SMMAs and zebrafish larvae, consisting of the electromagnetic coil array, power supply, circuit system, data acquisition system (DAQ), camera, and computer-based monitoring system.

The fabrication of the SMMA involved a sequential molding, material filling, curing, releasing, and magnetization process. Initially, S-shaped mold cavities were prepared using precision micro-milling, followed by filling with the NdFeB magnetic particle–PDMS composite mixture, as illustrated in Figure 2(Ai,Aii). An additional PDMS layer was subsequently introduced, and the excess material was removed to maintain the defined SMMA geometry (Figure 2(Aiii,Aiv)). The filled molds were thermally cured at 85 °C, after which the fabricated SMMAs were released from the mold and magnetized to establish their magnetic polarity (Figure 2(Av–Avii)). Detailed material compositions and fabrication procedures are provided in Experimental Section 2.1. Further, the experimental platform employed for SMMA actuation and behavioral observation is presented in Figure 2B. The system consisted of an electromagnetic coil array, a programmable circuit system, a power supply, a data acquisition system (DAQ), and a camera-based monitoring system. The microfluidic device was positioned above the electromagnetic coil array, and the electrical input to the individual coils was regulated through the control system to enable programmable magnetic actuation of the SMMAs. Simultaneously, the camera-based imaging system was employed for real-time observation and recording of SMMA motion and zebrafish larval responses within the microfluidic device. Detailed descriptions of the electromagnetic actuation system and imaging configuration are provided in Experimental Section 2.2 and Section 2.3. The spatially selective actuation strategy employed to independently control the SMMAs and generate localized hydrodynamic fields is described in the following section.

### 3.2. Programmable Actuation of Microactuators and Induced Flow-Mediated Behavioral Modulation

**Figure 3:**
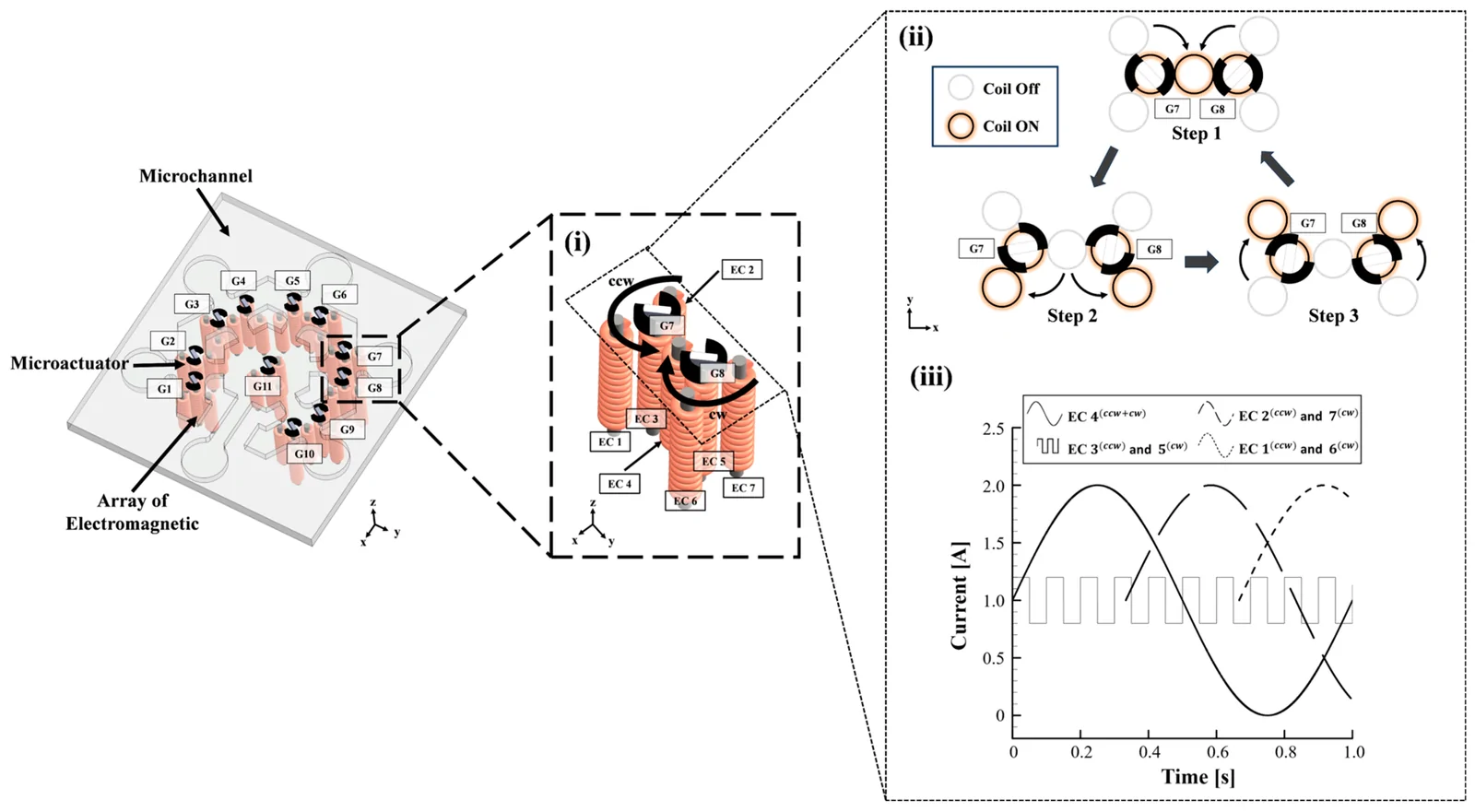
Electromagnetic actuation architecture for spatially selective control of SMMAs. (**i**) Schematic illustration of the microfluidic device integrated with an array of individually addressable electromagnetic coils (EC1–EC11) positioned beneath the chamber, enabling localized magnetic field generation for independent microactuator actuation. The spatial arrangement of coils relative to guidance microactuators (G1–G11) is indicated. (**ii**) Stepwise actuation sequence employed to generate controlled rotational motion under anchored conditions. Microactuators G7 and G8 were fixed in position by continuously energizing coils EC3 and EC5, while surrounding coils were sequentially activated to induce rotation. Counterclockwise rotation of G7 was achieved through sequential activation of EC2, EC1, and EC4, whereas clockwise rotation of G8 was achieved through sequential activation of EC7, EC6, and EC4, demonstrating directional control through programmable coil sequencing. (**iii**) The corresponding current supply required for realizing the mentioned motions of G7 and G8 was achieved using a phase-difference strategy.

The ability to generate controlled hydrodynamic motion is critical for eliciting mechanosensory responses in zebrafish larvae, since their behavioral adaptation is strongly governed by flow-based environmental cues. In this context, the development of a programmable and spatially controlled fluid environment was considered necessary to reliably trigger and regulate such responses within a microfluidic platform. To achieve this, a microrobotic actuation strategy was adopted, in which magnetically driven microactuators were utilized to produce localized and controllable flow fields. In this work, the microactuator was designed with an S-shaped geometry and was referred to as the SMMA, as introduced in the previous section. Further, to enable spatially selective actuation, an array of individually addressable electromagnetic coils was built and placed beneath the microfluidic chamber. Eventually, this facilitated independent control of each microactuator and subsequently enabled the generation of a localized magnetic field, as supported by the configuration shown in Figure 3. Meanwhile, a spatially fixed or anchored condition of the microactuator was considered essential to ensure that the generated hydrodynamic field remained localized and confined within the microfluidic environment, thereby preventing unintended translational motion that could disrupt controlled flow formation. To achieve this, certain coils in the electromagnetic coil array were continuously maintained in the energized state (ON state) to establish an anchoring condition, while the surrounding coils were sequentially activated to induce controlled rotational motion of the microactuator. In this process, selected coils were switched ON and OFF in a defined temporal sequence so that the magnetic field direction changed progressively and the embedded magnetic dipole within the microactuator continuously realigned with this field, resulting in controlled rotation. For example, in order to actuate and control the microactuators pair (G7 and G8) (Figure 3i), coils EC3 and EC5 were kept continuously energized to fix the position of these actuators, whereas neighboring coils (EC1, EC2, EC4, EC6, and EC7) were activated in a stepwise manner to generate rotational motions in different directions. In particular, microactuator G7 was required to rotate in the counterclockwise direction, and this motion was achieved through sequential activation of the surrounding coils. Starting from an initial orientation toward EC4 (step 1, as shown in Figure 3ii), coil EC2 was first activated (step 2), followed by EC1 (step 3) and then EC4, while the anchoring coil (EC3) remained continuously energized. This stepwise activation produced a progressive change in the magnetic field direction, resulting in controlled counterclockwise rotation of the microactuator. Similarly, for microactuator G8, which was required to rotate in the counterclockwise direction, coil EC7 was first activated (step 2, as shown in Figure 3ii) (starting from an initial orientation toward EC4), followed by EC6 (step 3) and then EC4, while the anchoring coil (EC5) remained continuously energized. As a result, bidirectional rotational motion control was achieved through programmable coil sequencing, which was critical since the direction of rotation determined the local flow structure around each microactuator. The corresponding supplied current in a phase-difference strategy for controlling the mentioned motions of G7 and G8 is shown in Figure 3iii). The details of the magnitude of the current supplied and the corresponding magnitude of the magnetic field generation are provided in the Experimental Section 2.2. ‘Electromagnetic Actuation System and Control.’ Furthermore, the rotational response of the SMMA was dependent on the applied actuation frequency. The angular velocity increased with increasing frequency and reached the maximum synchronized operating condition at 8 Hz, whereas a reduction in angular velocity was observed beyond 8 Hz due to the onset of unsynchronized rotational motion, as shown in Supplementary Figure S1. Therefore, 8 Hz was employed as the operating frequency to provide the maximum synchronized rotational response for generating the hydrodynamic stimuli used in the subsequent larval guidance experiments.

**Figure 4:**
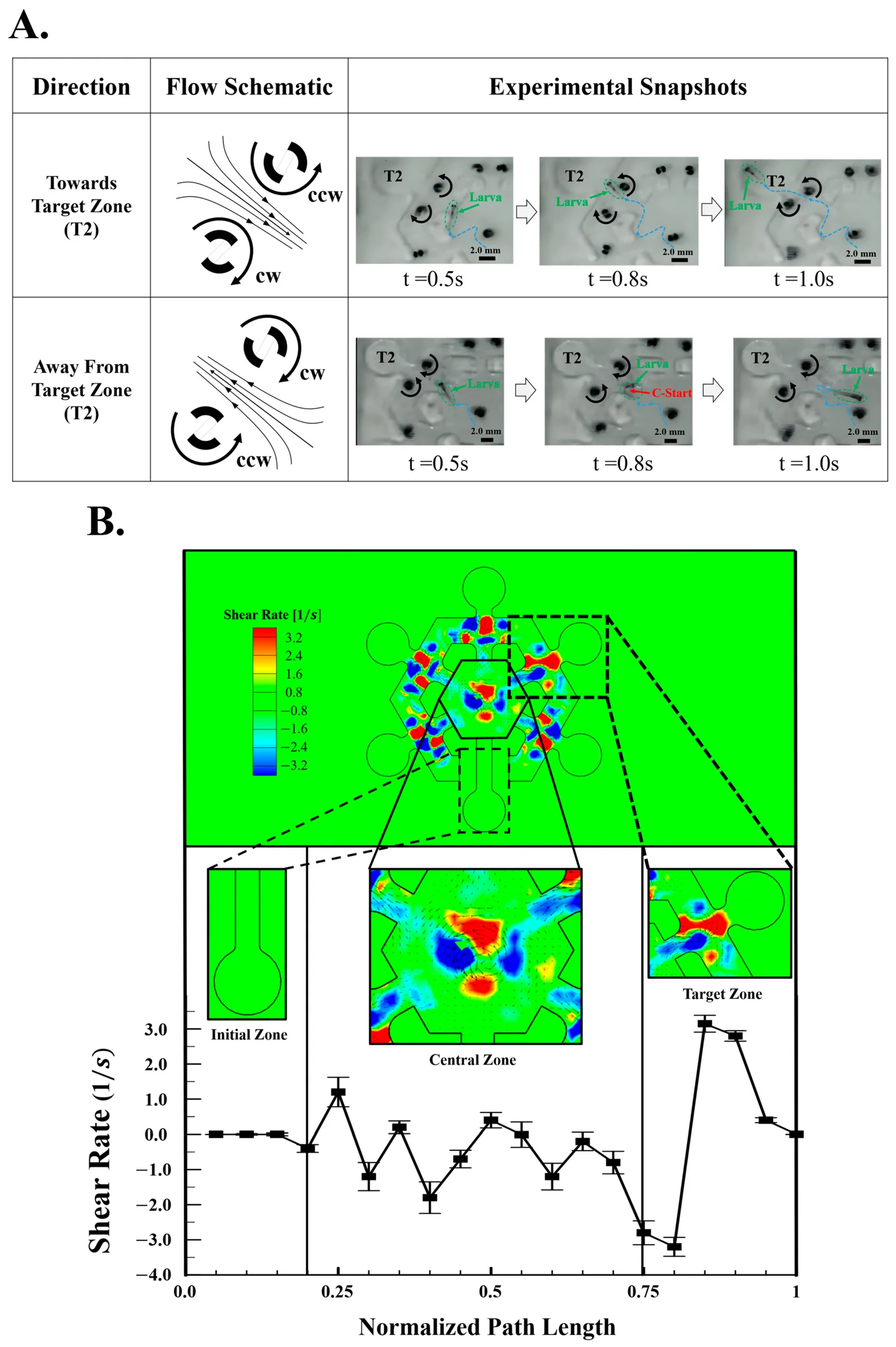
Flow-mediated larval guidance and quantitative characterization of the SMMA-generated hydrodynamic field. (**A**) Flow-mediated guidance mechanism and corresponding behavioral responses of zebrafish larvae generated through controlled rotational actuation of selected SMMA pairs. The rotational direction of the SMMAs regulated the direction of the induced flow relative to the intended larval movement. The experimental image sequences show the larval position, movement trajectory, direction of movement, designated target zone, and actuated SMMAs during the guidance process. The larval position is identified using green markers, while the movement trajectory is represented using blue markers. The characteristic C-start escape response is identified using a red marker. Rheotactic orientation was observed in response to the imposed flow, whereas exposure to the approaching flow stream induced a characteristic C-start escape reflex followed by rapid body reorientation and directional swimming. The corresponding markers indicate the progression of larval movement and the observed behavioral responses during flow-mediated guidance. The arrows under ‘Flow Schematic’ indicate the direction of the fluid flow induced by the SMMA. (**B**) Quantitative characterization of the generated hydrodynamic field using micro-particle image velocimetry. The spatial distribution of the induced flow and shear rate is presented along the larval transportation pathway from the initial region toward the designated target zone. Representative flow fields at different locations demonstrate the localized hydrodynamic disturbance generated by the rotating SMMAs and the corresponding variation in flow direction and shear rate along the transportation pathway. The arrows indicate the direction of the fluid flow induced by the SMMA.

The flow-mediated transportation of the zebrafish larva was initiated by the primary microactuator positioned near the initial zone. The localized hydrodynamic disturbance generated through its rotational motion induced the larva to leave its quiescent state and enter the central transport region, where subsequent directional guidance was achieved through the downstream guidance microactuators. Subsequently, directional control of larval movement was achieved through coordinated actuation of paired microactuators located at each target branch, which formed the fundamental mechanism for selective guidance in this study. In this configuration, a pair of guidance microactuators was positioned at the entrance of each target zone, and their combined actuation was utilized to either promote or suppress larval entry into that specific pathway. The key aspect of this mechanism lies in the fact that the resulting fluid motion generated by the paired microactuators determined whether the larva was guided toward or away from the selected target region (Figure 4A). For example, to transport the zebrafish into target zone T2, the corresponding guidance microactuators G3 and G4 were actuated with specific rotational directions (Figure 4A, towards T2). In this case, G3 was operated in the clockwise direction while G4 was actuated in the counterclockwise direction, such that the combined hydrodynamic interaction produced a net fluid motion directed away from the entrance of T2. Under this condition, due to the inherent flow-oriented behavior of zebrafish, the larvae were observed to align against the imposed flow and actively swam toward the target zone, thereby enabling successful entry into T2. This behavior of larvae guidance towards T2 is shown in the representative time-sequence snapshots in Figure 4A. To provide clear visualization of the guidance process, the larval position and movement trajectory were identified using green and blue markers, respectively, throughout the representative time-sequence snapshots in Figure 4A. These visual markers enabled the progression of larval movement relative to the designated target zone and the actuated SMMAs to be directly followed during the guidance process. In contrast, when the rotational directions of the paired microactuators were reversed, the resulting fluid motion was directed toward the entrance of the target zone, under which the larva exhibited a rapid avoidance response and was guided away from the pathway, thereby preventing entry into that region. Notably, this avoidance behavior was observed to occur in the form of a characteristic C-start response, which is commonly associated with startle reactions in zebrafish larvae, and this behavior can be clearly identified in Figure 4A, where the larva moved away from T2 at t = 0.8 s. The characteristic C-start response was further identified using a red marker in Figure 4A to clearly distinguish the rapid escape response from the preceding larval movement. Therefore, by systematically choosing the rotational directions of the paired microactuators, the local hydrodynamic field could be dynamically reconfigured to either attract or repel the larva from a given branch. This selective control of larval navigation through flow-mediated behavioral modulation represented a key feature of the present system and provided a robust mechanism for directing zebrafish movement in a programmable manner, which was essential for inducing controlled training behavior and enabling subsequent evaluation of learning and memory-related responses. In addition, the generated hydrodynamic field was quantified using the micro-particle image velocimetry (µPIV) technique, through which the spatial distribution of shear rate within the microchannel was obtained, as presented in Figure 4B. A detailed discussion on the µPIV experimental setup is provided in the Experimental Section 2.4. The ensemble-averaged shear-rate contours revealed that the flow field was primarily governed by the actuation of the microactuators, where each region of the channel exhibited distinct hydrodynamic characteristics depending on the local actuation condition. In the initial zone, the shear rate can be seen to remain negligible since no microactuator was positioned in this region, indicating the absence of externally induced flow. In contrast, within the central region where the primary microactuator was located, the shear rate was observed to increase significantly and fall within the range of 1.1 ± 0.05 to 3.2 ± 0.07 s^−1^, confirming that the primary microactuator generated sufficient hydrodynamic disturbance to initiate larval motion. Furthermore, in the vicinity of the target zone entrance, the shear rate can be observed to reach 3.2 ± 0.07 s^−1^ again, together with a corresponding change in flow direction that was oriented either toward or away from the target zones, depending on the rotational directions of the paired microactuators. These observations indicate that the primary microactuator was responsible for initiating the flow disturbance, whereas the paired guidance microactuators played a dominant role in shaping the local flow direction required for directional control. The corresponding variation in shear rate along the transport pathway is further illustrated in Figure 4B, where the distribution was evaluated along a normalized path length extending from the initial zone to the target region. Representative flow contours corresponding to specific regions along this path are highlighted in the inset images, which correspond to the initial, central, and target zone regions, respectively. Notably, the flow direction near the target zone was strongly influenced by the combined actuation of the paired guidance microactuators. For example, in the demonstrated case where target zone T4 was selected, the corresponding microactuator pair was actuated such that the resulting net flow direction was oriented away from T4. In contrast, for the other target zones, the flow direction was observed to be oriented toward their respective entrances, as shown in Figure 4B. This difference in flow orientation highlighted the ability of the system to dynamically reconfigure the local hydrodynamic field through selective actuation of microactuator pairs. In addition to the shear-rate distribution, the ensemble-averaged velocity magnitude and vorticity fields were quantified to further characterize the spatial distribution and rotational features of the induced flow, respectively, as presented in Supplementary Figure S2A,B. The ensemble-averaged velocity magnitude reached a maximum value of 38 mm s^−1^, demonstrating the localized fluid motion generated through rotational SMMA actuation. Similarly, the ensemble-averaged vorticity magnitude reached a maximum value of 2.1 s^−1^, confirming the localized rotational characteristics of the induced flow field. These velocity and vorticity distributions were consistent with the spatial variations observed from the shear-rate analysis and provided complementary characterization of the hydrodynamic field generated by the rotating SMMAs. Therefore, the combined shear-rate, velocity, vorticity, and flow-direction analyses confirmed that the microactuator system not only generates localized hydrodynamic disturbances but also enables precise control over the direction of fluid motion, which was essential for guiding zebrafish larvae toward or away from specific target zones.

### 3.3. Transportation Performance and Swimming Kinematics of Zebrafish Under Microactuator-Assisted Conditions

**Figure 5:**
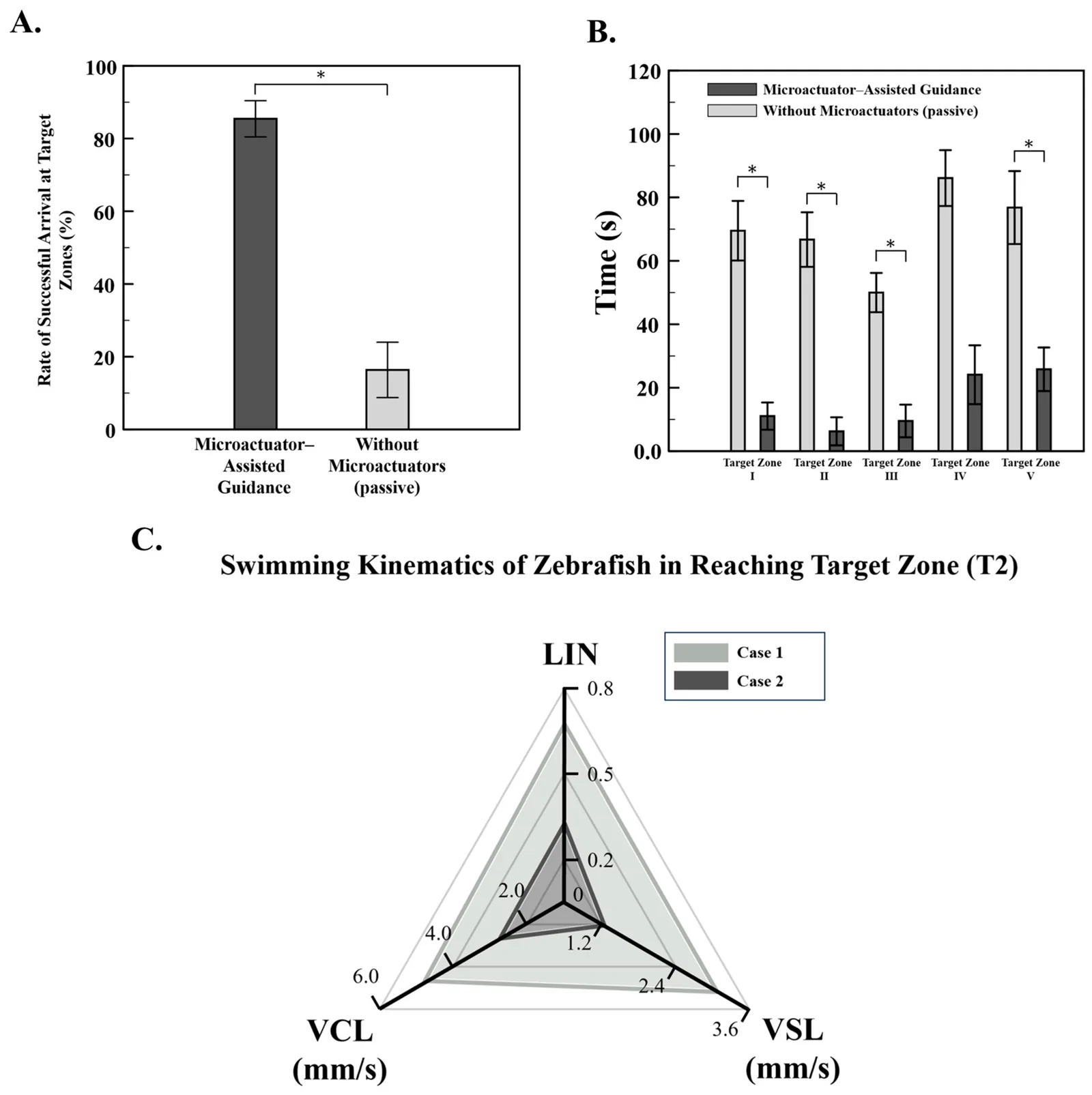
Quantitative evaluation of hydrodynamic guidance performance and swimming kinematics of zebrafish larvae under microactuator-assisted conditions. (**A**) Comparison of the cumulative success rate in reaching the assigned target zones under microactuator-assisted guidance and control (without microactuator (passive)) conditions. The success rate was defined as the percentage of larvae successfully reaching the designated target zone, aggregated across all target zones T1 to T5, with eight independent trials conducted for each case. A significant increase of 61.9% was observed under microactuator-assisted conditions, with the success rate reaching 81.2 ± 5.5%, whereas the control condition yielded a lower value of 19.33 ± 8.5%. Error bars represent the standard deviation obtained from three repeated trials. (**B**) Transport time required for zebrafish larvae to reach each target zone under control and microactuator-assisted conditions. A significant reduction in transport time was observed across all target zones under microactuator-assisted guidance compared to the control condition. The degree of reduction varied among the target zones, with higher improvements observed in T1 and T2 and relatively lower improvements in T5. Error bars represent the standard deviation obtained from three repeated trials. Statistical significance was evaluated using an independent-sample *t*-test, and significant differences are indicated by the symbol (*) representing *p* < 0.05. (**C**) Comparison of swimming kinematics of zebrafish larvae under two actuation conditions, namely activation of all microactuators (Case 1) and selective activation of only the microactuators corresponding to the target zone (Case 2), demonstrated for target zone T2. Under Case 1, higher values of VCL of about 5.0 mm/s and VSL of about 3.5 mm/s were observed, along with a higher LIN value of about 0.7, indicating more directed and efficient motion. In contrast, under Case 2, reduced values of VCL of about 2.0 mm/s and VSL of about 1.2 mm/s were observed, together with a lower LIN value of about 0.35, indicating less directed motion.

A systematic evaluation of flow-mediated transport behavior is essential to assess the effectiveness of externally induced hydrodynamic cues in guiding zebrafish larvae within confined microfluidic environments. To validate whether the introduced microactuator system was capable of guiding zebrafish larvae toward the designated target zones, it was necessary to distinguish its effect from the inherent influence of the microchannel geometry. In certain cases, the structural layout of the microfluidic network itself may bias larval movement toward specific pathways. Therefore, a comparative evaluation was carried out between a microactuator-assisted condition and a control condition in which all microactuators were maintained in the inactive state, and larval movement was monitored under identical experimental conditions. To quantify this effect, the rate of successful arrival at the target zone was evaluated. For each target zone, eight independent trials were conducted using different larvae, and this procedure was repeated across all target zones (T1 to T5). The resulting data were aggregated to obtain a cumulative success rate, which is presented in Figure 5A and Supplementary Video S1. An increase of 61.9% in the successful arrival rate was observed when microactuator-assisted guidance was employed compared to the control condition. In particular, it was observed that under microactuator-assisted guidance, the success rate reached 81.2 ± 5.5%, whereas in the control condition, the value was significantly lower at 19.33 ± 8.5%, thereby confirming that the introduced microactuator-induced hydrodynamic manipulation played a dominant role in directing larval movement.

Following this validation, it was further necessary to identify which target zones were naturally favored or less preferred by the larvae, since this distinction is critical for designing effective training and memory quantification experiments. In particular, training toward a less preferred pathway may provide a more rigorous condition for evaluating learning behavior. To achieve this, the transport time required for larvae to reach each target zone was measured under both conditions while maintaining the same number of larvae as described previously, and the results are presented in Figure 5B. A clear reduction in transport time was observed under microactuator-assisted conditions for all target zones. Specifically, for T1, the time can be seen to be decreased from 69.5 ± 9.4 s to 6.6 ± 2.5 s, corresponding to a reduction of 90.5%. Similarly, for T2, the time taken was decreased from 66.7 ± 8.6 s to 3.8 ± 8.6 s, corresponding to a reduction of 94.3%. Again, for T3, the time decreased from 50.0 ± 6.2 s to 5.7 ± 3.0 s, representing a reduction of 88.6%. For T4, the reduction from 86.1 ± 8.8 s to 14.2 ± 5.4 s corresponded to approximately 83.5%. For T5, the time decreased from 76.8 ± 11.5 s to 15.2 ± 4.0 s, corresponding to a reduction of 80.2%. These results indicate that although all pathways benefited from the introduced microactuator-induced hydrodynamic guidance, the degree of improvement varied depending on the target zone. In particular, T1 and T2 exhibited faster arrival and higher reductions, which may be attributed to more favorable alignment between the induced flow field and the natural swimming orientation of the larvae. In addition, this behavior may also be influenced by the specific rotational motion of the primary microactuator P, which could have facilitated more effective entry into these pathways. In contrast, T5 exhibited the highest transport time, indicating that it represented the most challenging pathway under the present configuration. It is suggested that this behavior may be influenced by the combined effects of the rotational direction of the primary microactuator and the spatial arrangement of the microfluidic network, which together define the local flow orientation experienced by the larvae. Based on this analysis, T5 was identified as the least preferred pathway, and thus selected as the most suitable candidate for subsequent training and memory quantification experiments.

To further understand the underlying mechanism governing larval guidance under different actuation strategies, the swimming kinematics were subsequently analyzed, as discussed in Figure 5C. In particular, a comparative study was performed to determine whether activating all microactuators within the system (Case 1) or selectively activating only the microactuators corresponding to the target zone (Case 2) entrance would provide more effective directional control. Such an evaluation was considered essential for identifying the most suitable actuation strategy for subsequent behavioral conditioning studies. To quantify the effect of these actuation conditions, swimming kinematic parameters were evaluated by employing the curvilinear velocity (VCL), the straight-line velocity (VSL), and the linearity index (LIN). The parameter VCL represents the actual swimming speed along the trajectory, VSL represents the effective displacement toward the target zone, and LIN, defined as the ratio of VSL to VCL, reflects the directional efficiency of motion, where values closer to unity indicate more linear and guided trajectories. These parameters were therefore employed to characterize the active swimming response and directional characteristics of the larvae under the imposed hydrodynamic conditions rather than to quantify passive displacement of the larvae with the surrounding fluid. For the purpose of demonstration, target zone T2 was considered, and the mentioned parameters were measured as shown in Figure 5C. Under Case 1, the corresponding guidance microactuators of T2, such as G3 and G4, were operated in clockwise and counterclockwise directions, respectively, to enable transport toward T2. Simultaneously, the remaining microactuator pairs that were placed at the other target zones (T1, T3, T4, and T5) were actuated in the opposite rotational configuration. That is, the microactuators G1, G5, G7, and G9 operated in the counterclockwise direction while G2, G6, G8, and G10 were actuated in the clockwise direction. In contrast, for Case 2, only the microactuators corresponding to T2, namely G3 and G4, were activated in clockwise and counterclockwise directions, respectively, while all other microactuators were maintained in the OFF state, thereby generating a localized flow field only near the target zone entrance. However, the primary microactuator P was kept active in both cases and rotated in the counterclockwise direction to generate the initial hydrodynamic disturbance required to initiate larval motion from the initial zone. The results showed that for Case 1, the larvae exhibited a higher VCL of about 4.74 ± 0.8 mm/s and a higher VSL of about 3.31 ± 0.6 mm/s, together with a LIN value of about 0.69 ± 0.05. In contrast, under Case 2, the VCL and VSL values were observed to decrease to about 1.21 ± 1.3 mm/s and 2.97 ± 1.3 mm/s, respectively, and similarly, the LIN value was also reduced to about 0.37 ± 0.3. The variation in these values may be associated with differences in the behavioral response of the larvae under the two actuation conditions. Specifically, in Case 1, it is suggested that the presence of flow cues generated from multiple microactuators, including those located at non-selected target branches, may have contributed to an earlier initiation of larval response. These distributed hydrodynamic cues could have reduced the exploratory time and facilitated a more immediate orientation of the larvae toward the transport pathway, thereby resulting in higher VCL, higher VSL, and improved directional alignment reflected by the higher LIN value. In contrast, in Case 2, where only the corresponding microactuator pair was activated, the absence of additional flow cues in other regions may have led to increased exploratory behavior before the larvae established a preferred direction. As a result, a delayed or less consistent response was seen, leading to reduced swimming activity and lower directional efficiency, as indicated by the decreased VCL, VSL, and LIN values. Based on these observations, it was suggested that activating all microactuators could provide a more effective strategy for guiding zebrafish larvae toward the selected target zone, as it produced stronger, more spatially distributed hydrodynamic interactions that eventually supported consistent, directed movement. Together with the rheotactic orientation and C-start escape response described in Section 3.2 (Figure 4A), these swimming characteristics further indicated that the observed larval transportation involved an active behavioral response to the imposed hydrodynamic stimuli rather than solely passive fluidic dragging or sweeping.

To further distinguish the observed behavioral response from possible secondary effects associated with electromagnetic actuation, a sham-control experiment was performed in which the electromagnetic field was applied while the SMMAs were maintained in an anchored state without rotational motion. Under this condition, the larvae exhibited natural exploratory behavior and moved throughout the microfluidic channel without consistent guidance toward any designated target zone, as demonstrated in Supplementary Video S2. Direct contact between the larvae and the stationary SMMAs was also observed, with the stationary microactuators functioning as passive physical obstacles during larval exploration. In contrast, target-directed navigation was observed during rotational SMMA actuation, indicating that electromagnetic actuation alone was insufficient to reproduce the behavioral response observed during flow-mediated guidance. In addition, possible local heating associated with electromagnetic actuation was evaluated by monitoring the temperature at different locations within the microfluidic channel over the complete actuation period from 0 to 120 s, as shown in Supplementary Figure S3A,B. The temperature remained consistent across the investigated locations, with a maximum difference of 0.21 °C measured throughout the actuation period. This limited thermal variation indicated that substantial local heating was not induced within the microfluidic environment under the employed electromagnetic actuation conditions. Thus, the results of sham-control and temperature measurements supported the association of the target-directed behavioral response with the hydrodynamic stimuli generated through rotational SMMA actuation rather than secondary electromagnetic or thermal effects.

### 3.4. The Characterization of Learning Behavior Associated with Microactuator-Assisted Hydrodynamic Guidance

**Figure 6:**
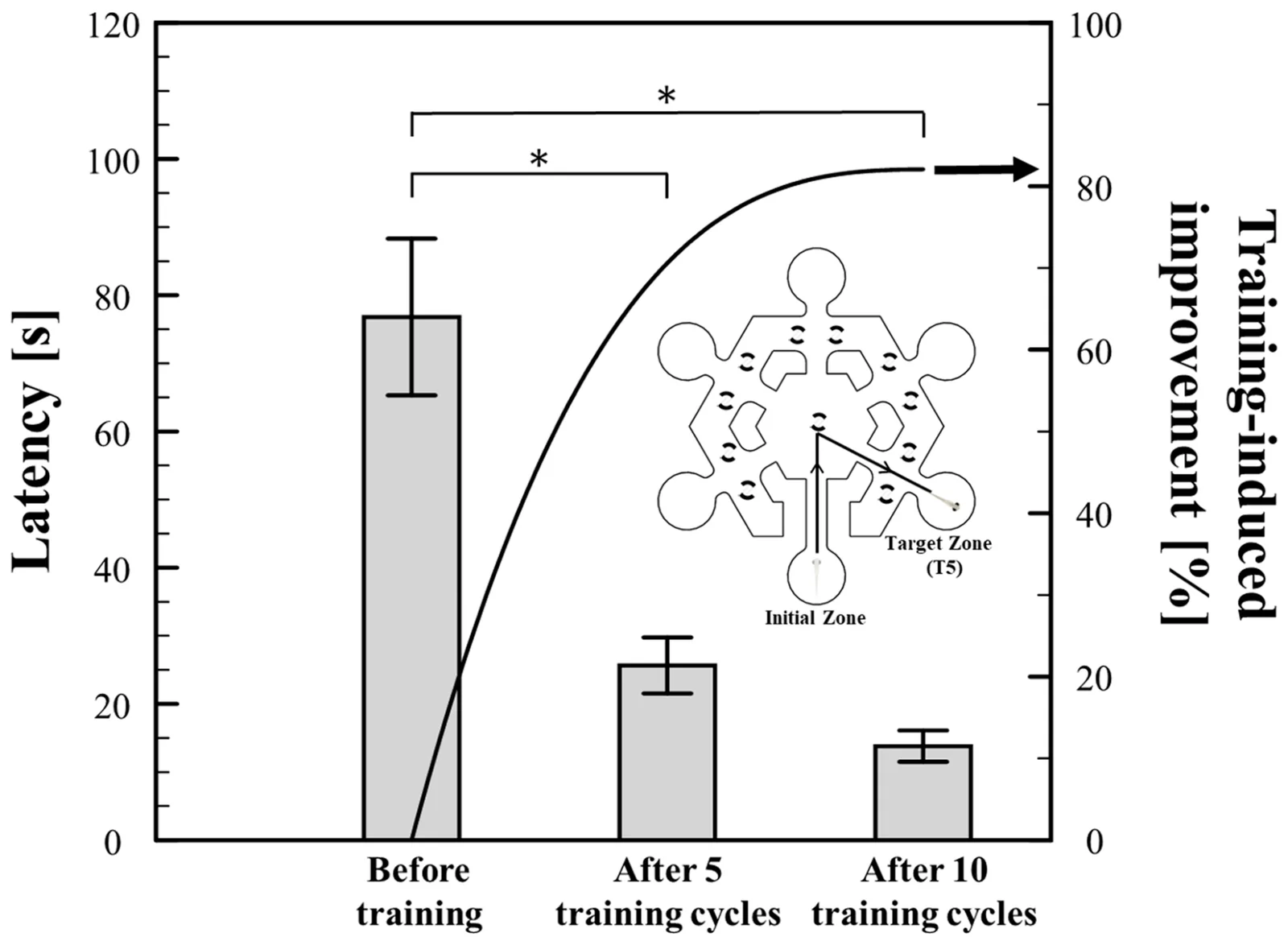
Quantification of learning behavior of zebrafish larvae under repeated hydrodynamic guidance. Latency was measured at target zone T5 under different training conditions, including pre-training and after multiple training cycles. The least preferred pathway, T5, was selected to minimize bias associated with naturally preferred navigation behavior. A progressive reduction in latency values can be observed with increasing training cycles, indicating improved navigation efficiency under repeated exposure to controlled hydrodynamic cues. Error bars represent the standard deviation obtained from three repeated measurements. Statistical significance was evaluated using one-way analysis of variance, and significant differences relative to the pre-training condition are indicated by the symbol (*) representing *p* < 0.05. An inset shows representative trajectories of zebrafish larvae moving toward target zone T5, highlighting the pathway along which latency was quantified.

Behavioral responses associated with navigation in aquatic organisms are primarily regulated by mechanosensory interactions with hydrodynamic cues, which provide essential directional information for movement within fluid environments. This underlying mechanism can be directly exploited in confined microfluidic systems, where spatially controlled flow fields can be generated to manipulate these mechanosensory inputs, thereby influencing the navigation behavior of zebrafish larvae. Such controlled modulation of flow-induced responses provides a suitable framework for inducing adaptive behavioral changes, which can be quantitatively evaluated by employing measurable parameters that reflect variations in navigation efficiency. In the present work, this adaptive response was assessed through the quantification of latency (Figure 6), defined as the time required for zebrafish larvae to reach a designated target zone under controlled hydrodynamic guidance conditions during repeated training cycles. To minimize bias toward naturally preferred navigation pathways, the least preferred target zone identified in the previous analysis, namely T5, was selected for this evaluation. A total of eight zebrafish larvae were employed, and the same eight larvae were individually followed from the pre-training assessment through all 10 training cycles. Therefore, the changes in this response time across the training cycles represented the progressive behavioral response of the same individual larvae rather than variability arising from different experimental groups. This longitudinal experimental design enabled the training-dependent behavioral response to be evaluated within the same individual larvae while considering differences in their initial responses. Given the limited sample size of eight larvae, the present behavioral assessment was considered a proof-of-concept evaluation of training-dependent behavioral adaptation using the proposed microrobotic microfluidic platform.

As illustrated in Figure 6, an inset depicting the representative movement of the larvae toward target zone T5 highlights the pathway along which the response time was quantified. Prior to training, a baseline latency of 77.6 ± 8.5 s was observed, indicating the inherent difficulty associated with navigating toward this pathway. With continued exposure to the controlled hydrodynamic conditions, a substantial reduction in the time required reach T5 was observed. Specifically, the latency decreased to 26.5 ± 5.4 s after 5 training cycles and further reduced to 13.8 ± 2.3 s after 10 training cycles. These reductions correspond to improvements of 66.2% and 82.1% relative to the pre-training condition. Furthermore, statistical analysis was performed using one-way analysis of variance, and it was confirmed that the reductions observed after both training intervals were significant when compared to the pre-training condition, as indicated by the symbol (*) representing *p* less than 0.05. In terms of the absolute change, a reduction of 51.1 s in the measured response time was observed from the pre-training condition to the fifth training cycle, whereas a smaller reduction of 12.7 s was observed from the fifth to the tenth training cycle. The reduced magnitude of change during the later training cycles indicated that the improvement in the behavioral response became progressively less pronounced with continued training. Based on this observed trend, additional training beyond 10 cycles was expected to produce progressively smaller changes in the measured response time. Therefore, 10 training cycles were selected as a reasonable endpoint for the present training protocol while avoiding unnecessary additional stimulation of the larvae. This progressive decrease in the time required to reach T5 indicates that the larvae were able to adapt their navigation behavior through repeated exposure to the imposed flow cues. At the beginning, higher latency values were observed, indicating increased exploratory behavior and a delayed response. In contrast, reduced response times observed during later stages of training were associated with improved orientation and a faster response to the hydrodynamic cues. Thus, the training-dependent reduction in latency demonstrated a progressive modification of the larval behavioral response toward T5 following repeated exposure to the controlled hydrodynamic stimulus. In addition, the gradual improvement observed across successive training cycles indicated that the behavioral adaptation developed progressively rather than instantaneously under repeated hydrodynamic stimulation. Meanwhile, it is suggested that this improvement may be associated with enhanced sensitivity of the mechanosensory system, enabling more efficient detection and interpretation of the imposed flow fields.

To further evaluate whether the behavioral response acquired during repeated training persisted following completion of the training process, an additional post-training retention assessment was performed by employing the same eight larvae. Following completion of the 10th training cycle, the SMMA was removed, and retention of the trained behavioral response was evaluated over a period of 15 min using a memory-extinction parameter termed freezing, as shown in Supplementary Figure S4. The freezing parameter was quantified based on the response time measured at each post-training time plot relative to the latency obtained at the end of the 10th training cycle and the corresponding pre-training latency. Freezing values of 85.6 ± 8.5%, 79.3 ± 8.0%, 62.7 ± 7.5%, 48.2 ± 4.5%, and 36.8 ± 3.5% were obtained at 3, 6, 9, 12, and 15 min, respectively. The freezing response progressively decreased with increasing post-training duration, resulting in a 57% reduction at 15 min compared with the response measured at 3 min. The initially high freezing response indicated that the behavioral response acquired through repeated training persisted following removal of the SMMA, whereas the subsequent time-dependent reduction demonstrated gradual extinction over the investigated period. Therefore, the post-training assessment demonstrated short-term retention followed by progressive extinction of the trained behavioral response. Nevertheless, further studies employing a larger number of larvae would be required to establish the observed behavioral response across a broader population and to evaluate inter-individual variability in greater detail. These findings demonstrate that the proposed microactuator-enabled hydrodynamic platform is capable of inducing training-dependent behavioral adaptation with measurable short-term retention in zebrafish larvae and provides a robust and non-contact approach for studying flow-mediated behavioral adaptation in microfluidic environments.

## 4. Conclusions

In this study, a programmable microfluidic assay was developed to generate spatiotemporally varying hydrodynamic environments by employing multiple SMMAs. Through this system, flow-mediated mechanosensory stimulation of zebrafish larvae was achieved. Further, the independent actuation of these SMMAs enabled the generation of localized vortical flow fields, which were effectively utilized for controlled, non-contact transportation of larvae toward designated target zones within the microfluidic platform. Meanwhile, the underlying flow dynamics responsible for this behavior were characterized using µPIV, through which the velocity, shear rate, and vorticity characteristics of the generated hydrodynamic fields were quantified. Subsequently, the effectiveness of the proposed system was first validated through transportation experiments, where a significant improvement in transport efficiency was observed under microactuator-assisted guidance when compared to the control condition. Furthermore, the influence of hydrodynamic actuation on larval swimming behavior was systematically evaluated by quantifying key swimming kinematic parameters, including curvilinear velocity, straight-line velocity, and linearity index. These parameters were employed to assess the effectiveness of different actuation strategies in guiding larvae toward target zones, where the results indicated that coordinated actuation of multiple microactuators facilitated improved directional movement and reduced exploratory behavior. Through these analyses, the most and least preferred pathways within the microfluidic network were identified based on navigation efficiency and transport time. In particular, the least preferred target zone was selected for subsequent behavioral evaluation to minimize inherent navigation bias and to establish a more stringent condition for assessing adaptive responses. Under this condition, repeated hydrodynamic guidance was applied as a training mechanism, and the resulting training-dependent behavioral adaptation was quantified using latency as a measure of navigation efficiency. A progressive reduction in latency was observed with increasing training cycles, indicating improved responsiveness and adaptation to the imposed flow cues. This observation suggests that the larvae were able to adjust their navigation behavior through repeated exposure to the hydrodynamic environment, reflecting an enhanced ability to interpret and respond to mechanosensory stimuli. Furthermore, the post-training retention assessment demonstrated that the acquired behavioral response persisted for a short duration following completion of training and progressively diminished over time, indicating short-term behavioral retention followed by gradual extinction. These findings demonstrate that the proposed microactuator-enabled platform was capable of both precise flow-induced manipulation and quantitative evaluation of training-dependent behavioral adaptation and short-term behavioral retention in zebrafish larvae.

## Data Availability

The data that support the findings of this study are available from the corresponding author upon reasonable request.

## Supplementary Materials

The following supporting information can be downloaded at https://www.mdpi.com/article/10.3390/bios16090480/s1. Figure S1. Frequency-dependent rotational response of the S-shaped magnetic microactuator (SMMA). Figure S2A,B. Ensemble-averaged hydrodynamic characteristics generated by rotational actuation of the S-shaped magnetic microactuators. Figure S3A,B. Temperature variation within the microfluidic channel during electromagnetic actuation. Figure S4. Temporal variation in freezing behavior of zebrafish larvae following repeated hydrodynamic training. Video S1. SMMA-induced transport of zebrafish larvae toward designated target zones. Video S2. Sham-control experiment demonstrating zebrafish larval behavior in the presence of an anchored SMMA without rotational actuation.
