# Supplementary material for: Behavioral Dynamics of Zebrafish Under Hydrodynamic Stimuli Induced by Magnetic Microactuator in Microfluidics
Source: Biosensors (Basel). 2026 Sep 1;16(9):480. doi: 10.3390/bios16090480 (PMC13604604; doi:10.3390/bios16090480)
Supplement: Supplementary file 1 [file biosensors-16-00480-s001.zip › Supplementary Figures.pdf]

Supporting Information

Behavioral Dynamics of Zebrafish Under Hydrodynamic Stimuli Induced by Magnetic Microactuator in Microfluidics

Dineshkumar Loganathan [1,†], *Pu-Hsiang Wang* [1,†], *and Chia-Yuan Chen* [1,2,*]

This supporting information file includes:

Supplementary Figures

Figure S1. Frequency-dependent rotational response of the S-shaped magnetic microactuator (SMMA).

Figure S2. Ensemble-averaged hydrodynamic characteristics generated by rotational actuation of the S-shaped magnetic microactuators

Figure S3. Temperature variation within the microfluidic channel during electromagnetic actuation.

Figure S4. Temporal variation in freezing behavior of zebrafish larvae following repeated hydrodynamic training.

Figure S1

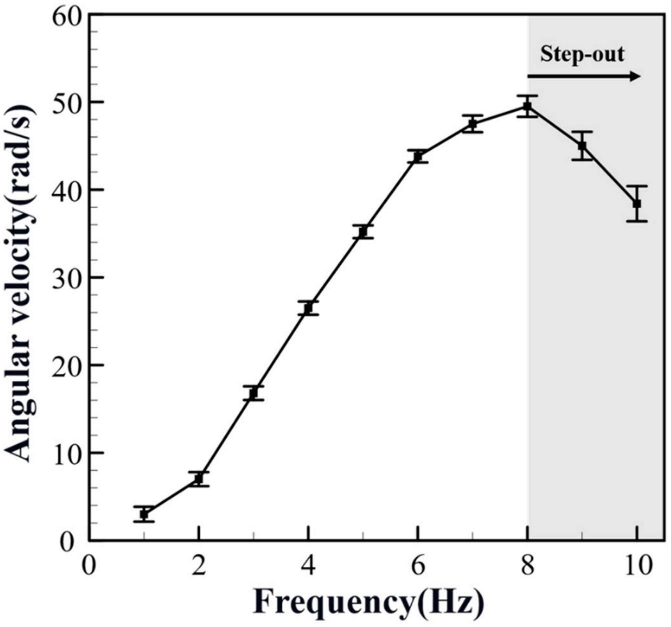

Figure S1. Frequency-dependent rotational response of the S-shaped magnetic microactuator (SMMA). The angular velocity of the SMMA was evaluated over an actuation frequency range of 1–10 Hz. The angular velocity progressively increased from 3.00 ± 0.85 rad/s at 1 Hz to a maximum of 49.50 ± 1.20 rad/s at 8 Hz, indicating an increase in rotational response with increasing actuation frequency. At frequencies greater than 8 Hz, step-out behavior was observed, with the angular velocity decreasing to 45.00 ± 1.60 rad/s at 9 Hz and 38.40 ± 2.00 rad/s at 10 Hz due to loss of synchronized rotational motion. Therefore, 8 Hz was selected as the operating frequency for the subsequent hydrodynamic stimulation experiments. Data are presented as mean ± standard deviation obtained from three repeated experiments.

Figure S2

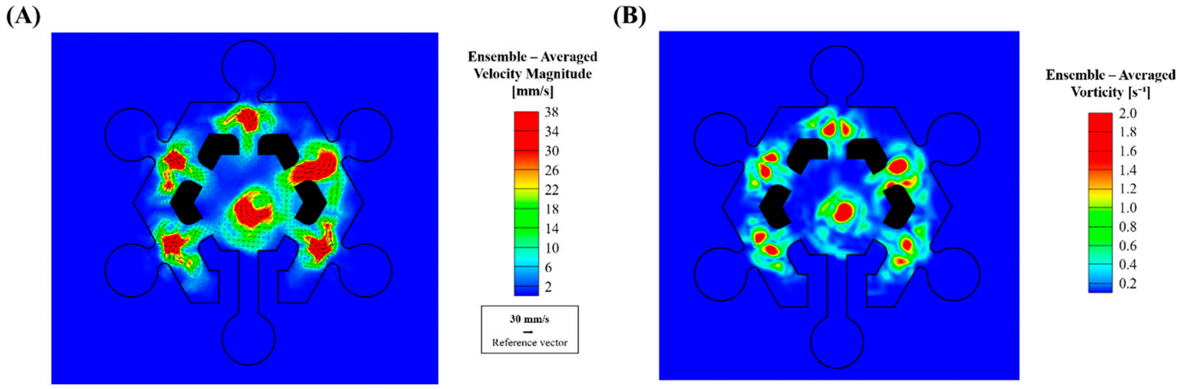

Figure S2. Ensemble-averaged hydrodynamic characteristics generated by rotational actuation of the S-shaped magnetic microactuators. (A) Ensemble-averaged velocity field obtained using micro-particle image velocimetry showing the spatial distribution and direction of the flow generated by the rotationally actuated SMMAs within the microfluidic chamber. The velocity vectors indicate the local flow direction, with the reference velocity vector corresponding to 30 mm/s, while the contour represents the corresponding velocity magnitude. A maximum ensemble-averaged velocity magnitude of 38 mm/s was observed in the regions influenced by SMMA rotation. (B) Corresponding ensemble-averaged vorticity field showing the spatial distribution of rotational flow structures generated within the microfluidic chamber. Localized regions of increased vorticity were observed around the actuated SMMAs, with a maximum vorticity magnitude of 2.1 s^{-1}, confirming the formation of localized vortical flow structures during rotational actuation.

Figure S3

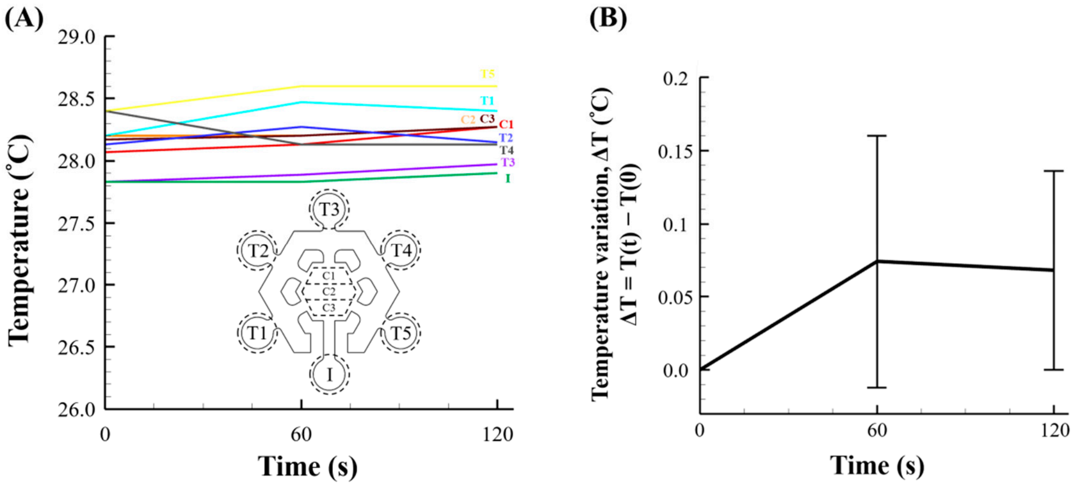

Figure S3. Temperature variation within the microfluidic channel during electromagnetic actuation. (A) Temperature measured at different locations within the microfluidic channel at 0, 60, and 120 s under the electromagnetic actuation conditions employed in the experiments. The central region of the microfluidic channel was divided into C1, C2, and C3, represented by red, orange, and brown lines, respectively. The peripheral regions consisted of the initial zone I and target zones T1 to T5, represented by green, cyan, blue, purple, gray, and yellow lines, respectively. The inset illustrates the corresponding measurement locations within the microfluidic channel. The temperature remained relatively stable across the investigated locations during the 120 s actuation period, with a maximum temperature difference of 0.21 °C. **(B)** Mean temperature variation across the different regions of the microfluidic channel at 0, 60, and 120 s. For each measurement location, the temperature variation was determined relative to its corresponding initial temperature at 0 s. The temperature variations obtained from the nine measurement locations, including I, T1 to T5, and C1 to C3, were then averaged at each time point. The mean temperature variations were 0 °C at 0 s, 0.074 °C at 60 s, and 0.068 °C at 120 s, with corresponding standard deviations of 0, 0.086, and 0.068 °C, respectively. The error bars represent the standard deviation of the temperature variations among the different spatial locations within the microfluidic channel. The small mean temperature variations and the absence of a further increase from 60 to 120 s indicate that no progressive temperature accumulation occurred during the investigated actuation period.

Figure S4

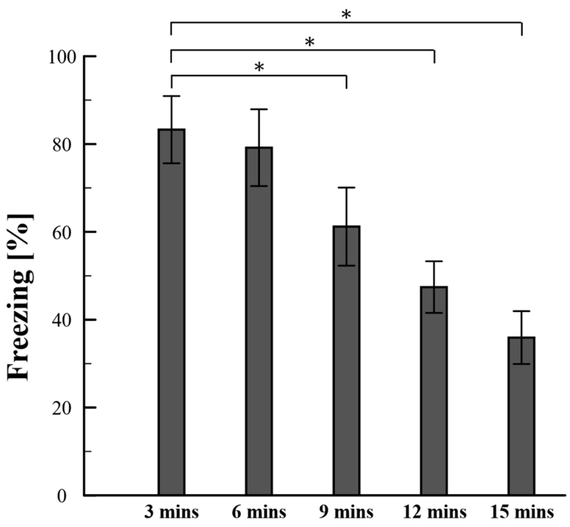

Figure S4. Temporal variation in freezing behavior of zebrafish larvae following repeated hydrodynamic training. Freezing behavior was evaluated at 3 min intervals for 15 min following completion of the 10th training cycle and removal of SMMA-mediated hydrodynamic stimulation. The same eight zebrafish larvae employed throughout the training experiments were followed during the post-training assessment. The freezing response progressively decreased from 85.6 ± 8.5% at 3 min to 79.3 ± 8.0% at 6 min, 62.7 ± 7.5% at 9 min, 48.2 ± 4.5% at 12 min, and 36.8 ± 3.5% at 15 min. The freezing response decreased by approximately 57% from 3 to 15 min, indicating a progressive reduction in the behavioral response over the post-training observation period. Data are presented as mean ± standard deviation for eight zebrafish larvae. Statistical significance among the different post-training time points was evaluated using one-way analysis of variance. Significant differences relative to the 3 min condition are indicated by the symbol (*) representing $p < 0.05$.
